# Seed mixes of intermediate grass:forb ratio create vegetation of greater ecosystem service provision in restorations

**DOI:** 10.1002/eap.70299

**Published:** 2026-09-08

**Authors:** Nathan Soley, Simone Schmidt, Brian Wilsey

**Affiliations:** ^1^ Department of Ecology, Evolution and Organismal Biology Iowa State University Ames Iowa USA

**Keywords:** ecosystem services, forb seed density, grass:forb seeding ratio, grassland restoration, pollinator habitat restoration, seed mix diversity, tallgrass prairie, trade‐offs

## Abstract

Restoring pollination services using forb‐dense seed mixes is seen as a possible way to mitigate global pollinator decline. Research is needed on whether this management factor impacts other ecosystem services that could be influenced by greater emphasis on forbs than grasses in seed mixes. We tested grass:forb seeding ratios varying from 0:100 to 100:0 for their effects on multiple ecosystem services in a prairie restoration. We hypothesized that intermediate grass:forb seeding ratios, which have greater plant diversity, would result in greater supporting, regulating, and aesthetic services. We sowed 11 grass:forb seeding ratios and measured ecosystem service indicators (plant richness, plant productivity, animal‐pollinated flower production, volunteer biomass, pollinator visitation, and butterfly visitation, an indicator of aesthetic services) over four growing seasons at a restoration in Iowa, USA. There were significant quadratic relationships between grass:forb seeding ratio and all of the above indicators except net primary productivity, which was not significantly related to grass:forb seeding ratio, suggesting no trade‐offs among these ecosystem services at intermediate ratios. We further showed that greater service provision was related to increases of various seed mixture diversity indices. A structural equation model showed seed mix diversity has positive effects on invasion resistance and animal‐pollinated flower production that indirectly enhance pollination and aesthetics. Volunteer biomass was suppressed with greater seed mix diversity. Overall, these results indicate that prairie restorations with intermediate grass:forb seeding ratios will have the optimal provision of ecosystem services, especially those that generate aesthetic appeal to people.

## INTRODUCTION

Ecosystem services are the benefits people obtain from ecosystem functions and provide either supporting, regulating, provisioning, or cultural services to society (Costanza et al., 1997; MEA, 2005). Supporting services are essential for the production of other ecosystem services, regulating services regulate ecosystem processes, and cultural services are nonmaterial benefits people obtain from ecosystems (MEA, 2005). In grasslands, these ecosystem services include forage production, carbon storage, pollinator provision, and recreation (Bengtsson et al., 2019). Grasslands have experienced greater habitat loss with less protection compared to other biomes despite the essential ecosystem services they provide (Bengtsson et al., 2019; Hoekstra et al., 2005; Schulte et al., 2017; Zhao et al., 2020). Plant diversity enhances ecosystem services when multiple years, sites, and environmental change contexts are considered (Isbell et al., 2011), and restoring native grassland diversity has become a global initiative for this reason (UNEP & FAO, 2020). In this study, we assess how multiple supporting, regulating, and cultural ecosystem services are affected by seed mix design in the tallgrass prairie system of the central United States, where pollinator decline is occurring (Burkle et al., 2013; Grixti et al., 2009). Increasing plant biodiversity for pollinator habitat creation is a common goal of prairie restorations (Barak et al., 2022), but research is needed on how this will affect other ecosystem services.

Pollinator decline and its threat to pollination services has become a global concern (Potts et al., 2010). Flower‐dense habitat in grassland restorations can help to mitigate pollinator decline (Blaauw & Isaacs, 2014; Borchardt et al., 2023; Goulson et al., 2015; Hopwood, 2008; Stein et al., 2020; Tonietto & Larkin, 2018), but restorations that provide more services may provide a greater variety of benefits to society (Liu et al., 2025; Wratten et al., 2012). The desire for multiple ecosystem services from the environment is common across stakeholder groups (Lamarque et al., 2011; Peter et al., 2022; Schulte et al., 2017). Here, we examine multiple ecosystem services from restored vegetation including that managed for abundant animal‐pollinated flower production.

Restoration practitioners often have to decide which species and ratios of functional groups to use in seed mixes, and the consequences of this decision on ecosystem services have not been investigated across the range of possible choices (Dickson & Busby, 2009). Forbs are broadleaf herbaceous plants that can produce animal‐pollinated flowers, and grasses do not produce animal‐pollinated flowers (Soley and Wilsey, 2026). Grass:forb seeding ratios of 0:100 are becoming more common with emphasis on pollinator habitat (Lybbert et al., 2022; Nichols et al., 2022; Schmidt et al., 2020). The cost of forb seed is a limiting factor in their planting over large scales (Simanonok et al., 2022). Grass:forb seeding ratios near 100:0 are most common because of their affordability and peoples' desire to have grassland restorations reflect the dominant vegetation type (Ye & Lu, 2025). Grass dominance can lower species diversity in tallgrass prairie (Grman et al., 2021; McCain et al., 2010; Sonkoly et al., 2019), suggesting a cost to other ecosystem services. We investigate whether variation in the relative abundances of grasses and forbs in seed mixes significantly affects ecosystem services impacted by pollinator habitat restoration.

Having a high proportion of forbs in seed mixes could positively affect supporting, regulating, and cultural ecosystem services in restorations (Dickson & Busby, 2009; Kaul & Wilsey, 2021; Klopf et al., 2014; Meissen et al., 2020; Müller et al., 2024; Soley & Wilsey, 2025). Forbs contribute more to overall species richness than grasses because forbs are more speciose than grasses (Bråthen et al., 2021; Collins & Steinauer, 1998; Dickson & Busby, 2009; Kaul & Wilsey, 2021). Most forb species offer nectar and pollen rewards to attract pollinators, and greater abundance of these forbs can result in greater animal‐pollinated flower production (Meissen et al., 2020; Müller et al., 2024). This flower production is a supporting service for pollinators. In turn, pollinators provide a regulating ecosystem service by maintaining animal‐pollinated plant reproduction (Artamendi et al., 2025; Fontaine et al., 2006; Lundgren et al., 2016; MEA, 2005; Travis & Kohn, 2023; Vázquez et al., 2005) and pollination to crops (Brittain et al., 2013; Garibaldi et al., 2013; Orford et al., 2016; Reilly et al., 2020; Sandhu et al., 2016). Pollinator diversity can enhance pollination services (Artamendi et al., 2025; Fontaine et al., 2006; Lemanski et al., 2022; Winfree et al., 2018). Wild bees and flower flies are important pollinators in grasslands (Fontaine et al., 2006; Heuel et al., 2024; Lucas et al., 2018; Oleques et al., 2021; Parrish & Bazzaz, 1979), with bees having been shown to be more strongly linked to changes in flower production (Blaauw & Isaacs, 2014; Hegland & Boeke, 2006; Williams et al., 2015). Animal‐pollinated flower production also increases the visitation of butterflies (Feber et al., 1996; Steffan‐Dewenter & Tscharntke, 1997), which are of interest to the general public because of their aesthetic qualities (Bullock et al., 2021; Habel et al., 2021; Lopez‐Callado et al., 2016). High production of showy flowers is another aesthetically attractive attribute of forb abundance (Graves et al., 2017; Kaul et al., 2023). Aesthetics is considered to be a cultural ecosystem service for its positive impact on societal well‐being (MEA, 2005), and aesthetics can heavily influence motivations for biodiversity conservation (Lundberg et al., 2019; Metrick & Weitzman, 1996).

Alternatively, having high grass abundance in seed mixes could also enhance other ecosystem services. Grasses have been shown to produce more biomass compared to forbs, especially below ground (La Pierre et al., 2011; Podzikowski et al., 2023; Smith & Knapp, 2003; Zhang et al., 2022). Higher grass abundance may provide greater forage to livestock and is a supporting ecosystem service in this sense (MEA, 2005). Also, high biomass production is correlated with competitive ability and, therefore, an increased potential to resist invasion by exotic weeds, which strongly predict plant diversity in grassland restorations (Kaul & Wilsey, 2021). Invasion resistance is a regulating ecosystem service (MEA, 2005). Grasses are important at resisting invasion in mixed grass‐forb mixtures (Davies et al., 2017; Eastburn et al., 2018; Fargione et al., 2003; Foster et al., 2002; Grman et al., 2021; Kiss et al., 2022; Meissen et al., 2020; Roscher et al., 2009; Smith et al., 2004; Török et al., 2010; Wilsey, 2010) but can increase invasion at very high levels of dominance (Losure et al., 2007). Indeed, grassland communities with higher plant diversity have been shown to be more resistant to invasion than grass‐only plantings (Jaksetic et al., 2018; Maron & Marler, 2007), so grassland restorations sown with intermediate grass:forb seed ratios are predicted to be most resistant to invasion.

A trade‐off may exist between increasing invasion resistance with grass seed abundance and increasing animal‐pollinated flower production with forb seed abundance. Trade‐offs may be less likely with greater seed mix diversity, which changes with grass:forb ratio (Table 1) and can positively affect ecosystem services. Indices such as taxonomic diversity (Simpson's *D*) and functional group diversity, which account for plant relative abundances, vary most with grass:forb seeding ratio, while species richness is based on the simple presence or absence of grass or forb species. Species richness has been manipulated to test for its effect on ecosystem services, which is generally positive (Cardinale et al., 2012; Feld et al., 2009; Lemanski et al., 2022). Greater species richness increases aboveground net primary productivity (ANPP) (Cardinale et al., 2007; Grace et al., 2016; Schmitz et al., 2013) and can increase pollinator richness (Ebeling et al., 2008; Hulting et al., 2024) and butterfly abundance (Myers et al., 2012). Evenness, or the proportional diversity among plant types, is less often manipulated to test for diversity effects on ecosystem services, but it has been shown to positively affect ANPP (Mattingly et al., 2007; Schmitz et al., 2013; Wilsey & Potvin, 2000) and invasion resistance (Losure et al., 2007; Smith et al., 2004; Wilsey & Polley, 2002). Thus, some ecosystem services may not trend linearly with grass:forb seeding ratio and instead may peak with higher diversity in between 0:100 and 100:0, where functional group diversity is higher (Wilsey, 2021). In a recent large‐scale study, remnant prairies had higher plant diversity but lower forage quality compared to low‐diversity exotic grasslands, revealing a trade‐off between these supporting services (Martin et al., 2014). In prairie restorations of the upper United States, trade‐offs were not found and instead plant diversity was positively related to animal‐pollinated flower production, which itself was positively related to ANPP (Zirbel et al., 2019). Zirbel et al. (2019) identified trade‐offs through correlations between ecosystem functions. We build upon this approach by conducting an experiment testing causal relationships between grass:forb seeding ratio and multiple ecosystem services and identifying any trade‐offs among services.

**TABLE 1:** The grass:forb seeding ratios, grass:forb seed mass ratios, density, and diversity of treatments.

Replicates (55 total)	Grass:forb based on seed numbers	Grass:forb based on seed mass	Functional group diversity	Grass seeds/m^2^ (feet^2^)	Forb seeds/m^2^ (feet^2^)	Species richness	Simpson's species diversity	Evenness
5	100/0	100/0	1.00	387.5 (36)	0 (0)	10	9.39	0.94
5	90/10	90.6/9.4	1.22	348.8 (32.4)	38.8 (3.6)	58	11.56	0.20
5	80/20	81.1/18.9	1.47	310.0 (28.8)	77.5 (7.2)	58	14.45	0.25
5	70/30	71.4/28.6	1.72	271.3 (25.2)	116.3 (10.8)	58	18.34	0.32
5	60/40	61.6/38.4	1.92	232.5 (21.6)	155.0 (14.4)	58	23.53	0.41
5	50/50	51.7/48.3	2.00	193.8 (18)	193.8 (18)	58	30.19	0.52
5	40/60	41.7/58.3	1.92	155.0 (14.4)	232.5 (21.6)	58	37.86	0.65
5	30/70	31.5/68.5	1.72	116.3 (10.8)	271.3 (25.2)	58	44.77	0.77
5	20/80	21.1/78.9	1.47	77.5 (7.2)	310.0 (28.8)	58	47.81	0.82
5	10/90	10.6/89.4	1.22	38.8 (3.6)	348.8 (32.4)	58	45.16	0.78
5	0/100	0/100	1.00	0 (0)	387.5 (36)	48	38.42	0.80

*Note*: The treatments were applied to the main plots in the experiment using a deWitt replacement design approach that keeps overall seeding density constant at 36 seeds/feet^2^ (387.5 seeds/m^2^). The 11 levels of grass:forb seeding ratios vary functional group diversity from 1 to 2.

**FIGURE 1:**
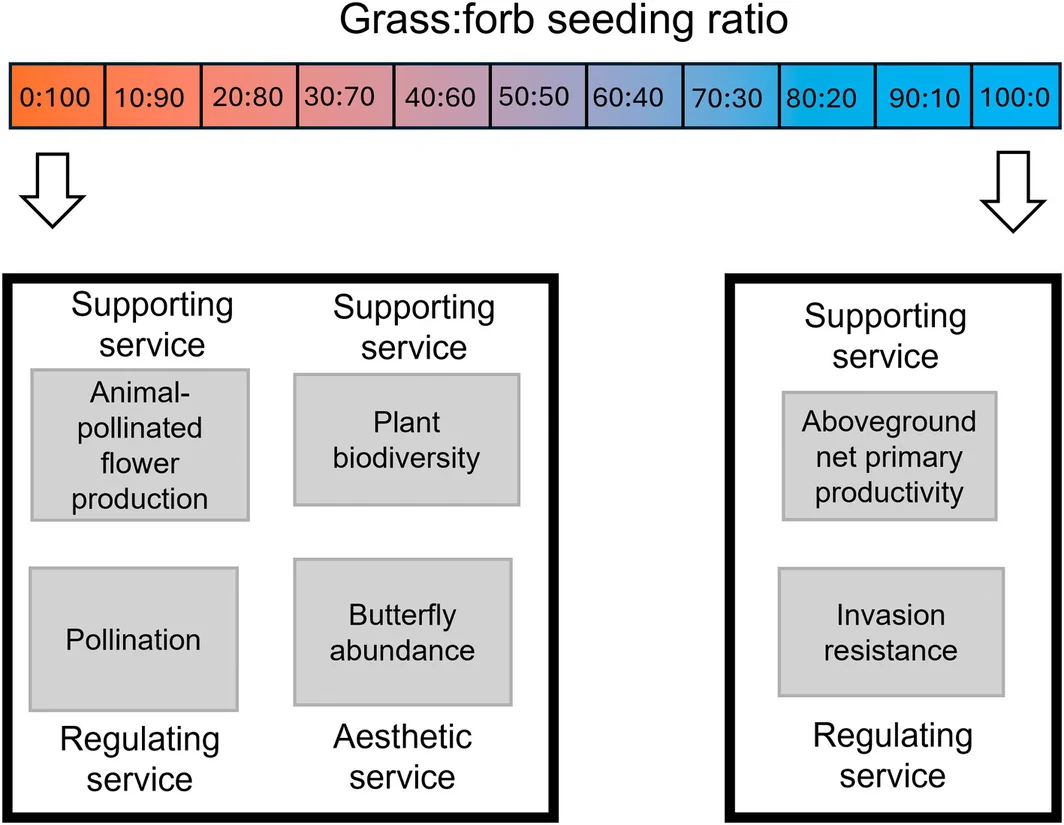
Diagram of the grass:forb seeding ratios tested in this experiment and the ecosystem services measured. The color red represents forb seed and blue represents grass seed. The arrows represent positive effects based on alternative hypotheses (see main text).

We tested seed mix grass:forb ratio effects on ecosystem services and trade‐offs for multiple ecosystem services. To do this, we planted different grass:forb ratios of a prairie seed mix and measured ecosystem services during and after establishment. Ratios ranged from 0:100 to 100:0, which allowed us to examine relationships with ecosystem services and whether diversity changes in these mixtures influence service delivery. Our hypothesis was that intermediate grass:forb seeding ratios would result in greater supporting, regulating, and aesthetic ecosystem services; the relationship between grass:forb seeding ratio and ecosystem services will be quadratic. Alternatively, there may be ecosystem service trade‐offs. Alternative hypotheses are that grass:forb seeding ratio will be linearly related to ecosystem services, due to (1) the increase in grasses, which would lead to a positive effect on services associated with ANPP, or (2) the decline in forbs, which would lead to a negative effect associated with pollinators (Figure 1). To test these hypotheses, we examined whether there was a significant quadratic, positively linear, or negatively linear relationship between grass:forb seeding ratio and each ecosystem service. We further examined which component of the grass:forb seeding ratio, that is functional group diversity, seed mix richness, evenness, Simpson's diversity, or forb seed density had the strongest effect on ecosystem service measures. Finally, we utilized factor analysis and a structural equation model (SEM) to further describe trade‐offs and/or synergies among ecosystem services.

## METHODS

### Study site

This study was carried out at a 20.8 ha site in central Iowa, USA (41°59′03.3″ N, 93°41′38.1″ W), during 2021–2024. The mean annual temperature is 9.3°C and the mean annual precipitation is 837 mm. Precipitation in the years of study were 753, 824, 815, and 875 mm in 2021, 2022, 2023, and 2024, respectively. Weather data were obtained from the Iowa Environmental Mesonet IATAME station report that is 4 km from the site (IEM, 2025). Soils are Mollisols with pH of 7.5–7.8. The site was in annual row crop production for many years before the study. Further site characteristics are detailed in Soley and Wilsey (2025).

### Experimental design

Eleven different grass:forb seeding ratios were assigned to experimental plots in a completely randomized design. The ratios ranged from 0:100 to 100:0 grass:forb seed differing at 10% increments; 100:0 grass:forb mixes had 10 perennial native graminoid species, 0:100 grass:forb mixes had 47 perennial and one biennial native forb species, and the nine other grass:forb mixes had these 58 species combined (Appendix S1: Table S1). The different grass:forb seeding ratios affected species richness, evenness, species diversity (Simpson's *D*), and functional group diversity of seed mixes (Table 1). Seed viability was tested and was over 90% for almost all species (Appendix S1: Table S1). Furthermore, we removed impurities such as weed seeds from each bag before the seed mixes were created, so we were using very close to pure live seeds. Seeds were mixed according to the different ratios and broadcast seeded into plots at a rate of 387.5 seeds/m^2^ in March 2020, a rate close to the seed rate recommended for our area (Meissen et al., 2020). Plots were 30 × 30 m and were separated by at least 5‐m alley‐ways into which a 50:50 mix was applied. There were five replicates of each grass:forb seeding ratio treatment (*n* = 55). The whole site was mowed twice to 15 cm during the first growing season to reduce weed pressure and burned in the first fall. No herbicide application or weeding was done in the experimental plots. Further details of the experimental design are in Soley and Wilsey (2025).

### Sampling

Ecosystem services of ANPP, animal‐pollinated flower production, invasion resistance, and butterfly visitation were sampled from the second to fourth growing season (2021–2023), and plant biodiversity and pollinator visitation were sampled from the second to fifth growing season (2021–2024). In this study, we classify plant biodiversity as a supporting ecosystem service because of its association with greater ecosystem service provision in restorations (Rey‐Benayas et al., 2009).

To test whether grass:forb seeding ratio affected the supporting services of plant biodiversity, ANPP, and animal‐pollinated flower production, we surveyed and harvested vegetation within grass:forb ratio plots. Plant biodiversity was measured as plant species richness, which we measured with “meandering” or “random” walks through each 30 × 30 m plot to record all plant species observed within 7 min during June of each year (Larson et al., 2020; Meissen et al., 2020; Sutton & Morgan, 2009; Wilsey et al., 2005). The whole plot was sampled to make it more consistent with the butterfly and pollinator sampling. To sample ANPP, a 0.1‐m^2^ quadrat was located randomly in a plot at peak biomass and all living vegetation within was clipped to 2 cm height, with one clip plot sample per grass:forb ratio plot in 2021, and two per plot in 2022 and 2023. Biomass was dried in ovens at 65°C for at least 24 h and weighed. Litter and standing dead vegetation were not included in our ANPP estimates, but both were close to zero during the study. To measure animal‐pollinated flower production, we estimated floral volume of the community per time interval within a growing season in three randomly placed 3.14‐m^2^ sampling areas in each grass:forb ratio plot. Floral volume is the volume (in cubic centimeters) of all animal‐pollinated flowers surveyed in the community (Soley & Wilsey, 2025). There were six time intervals each year (April–May, June, July, August, September, October) in which flowers were surveyed at least once but more often twice or more per interval.

To test whether grass:forb seeding ratio affected the regulating services of invasion resistance and pollination, we sampled volunteer species biomass and visiting bee and flower flies in the grass:forb ratio plots. Volunteer biomass was quantified by separating the clipped vegetation into seeded and volunteer species and recording volunteer species' biomass separately. Volunteer species included multiple invasive species such as Canada thistle (*Cirsium arvense*) that can reduce diversity in restorations in the area (Kaul & Wilsey, 2021; Martin & Wilsey, 2012), as well as some weedy native species (e.g., *Conyza canadensis* and *Ambrosia* spp.). All bees (Apoidea) and flower flies (Syrphidae) were observed for 5 min within a 3.14‐m^2^ sampling area within each plot during seven samplings across the 2021–2022 growing seasons (Soley & Wilsey, 2025) and on July 24–25 and September 7–10, 2023, and July 24–30, 2024. Bees and flower flies that came in contact with reproductive organs of a flower were recorded as visitors (Lázaro et al., 2013; Williams et al., 2015). Visitors were identified to genus in the field (Ebeling et al., 2008), and any that could not be identified were collected and later identified in the lab. Bee and flower fly taxonomic richness is based on genera richness.

To test whether grass:forb seeding ratio affected aesthetics services, we observed butterfly (Rhopalocera) visitors to all plants within grass:forb ratio plots during eight sampling rounds each year from the second to fourth growing season. Sampling occurred twice in June, July, August, and September. Our observations consisted of walking two diagonal paths across plots from opposite corners for 2 min, recording all butterflies seen. Observations were not done in especially windy weather (>15 km/h) or when it was raining.

### Analyses

ANPP, animal‐pollinated flower production, and volunteer biomass were averaged across subsamples per plot within a growing season to obtain one value per year. Butterfly abundance was summed across samplings per plot per year. Bee and flower fly abundance and richness were summed across all samples per plot because there were many zeroes when each year was included.

We analyzed the effect of grass:forb seeding ratio on pollination services using generalized linear models, and other ecosystem services were analyzed using linear mixed models, with grass:forb ratio, year, and their interaction as fixed effects. Mixed models were repeated measures analyses with the error term as grass:forb ratio replicate nested within grass:forb ratio. One 90:10 plot was not measured because it was mislabeled (for a total of 54 plots). Year was a repeated measures term in mixed models and was analyzed using a first‐order autoregressive covariance structure for plant richness, animal‐pollinated flower production, and butterfly abundance, while unstructured covariance was used for ANPP and volunteer biomass. To test linear and quadratic trends of grass:forb seeding ratio on each response, we used a priori polynomial contrasts by generating contrast coefficients of grass:forb ratios with Proc IML in SAS 9.4 (Little et al., 2006). We checked residuals of mixed models for normality and square root (√ + 1) transformed ANPP and volunteer biomass, log (ln + 1) transformed animal‐pollinated flower production, and cube root (∛ + 1) transformed butterfly abundance to meet assumptions of normality. Animal‐pollinated flower production is of seeded species, with the second and third growing season reported by Soley and Wilsey (2025), and the fourth growing season presented here. Analyses were carried out using “Proc Mixed” in SAS 9.4.

In models of bee and flower fly responses, grass:forb seeding ratio was the fixed effect. A negative binomial distribution with log‐link function was used to analyze bee and flower fly abundances because data were significantly overdispersed when a Poisson distribution was used. A Poisson distribution was used to analyze bee and flower fly richness. We verified that models were not overdispersed with χ^2^/df of ≤1. Bee and flower fly richness responses were further analyzed with abundance as a covariate to test if grass:forb ratio effects were independent of the number of individuals sampled. Analyses were carried out using “Proc Genmod” in SAS 9.4.

We further compared which components of our grass:forb seeding ratios (Table 1) affect ecosystem service provision using generalized linear models and Akaike information criterion (AIC). Generalized linear models tested the prediction that seed mix functional group diversity is most predictive of ecosystem service provision or if species richness, evenness, species diversity (Simpson's *D*), or forb density were significantly related to ecosystem services. We used a negative binomial distribution with a log‐link function to account for nonparametric distributions of predictors and overdispersion of responses. When more than one model varied significantly (*p* value < 0.05), the one with the lowest AIC value was considered the most probable to explain diversity effects. Analyses were carried out using “Proc Genmod” in SAS 9.4.

We used a factor analysis using correlations of variables with principal components (PC) to examine orthogonal components of variation and intercorrelations among diversity measures (functional group diversity, species richness, evenness, Simpson's diversity) and the ecosystem service measures described above to further test for trade‐offs. The number of factors to interpret was based on a scree plot, and three factors were above the “elbow” where eigenvalues dropped off. Ecosystem service loadings with a high *r* value and opposite signs along a PC axis were interpreted to indicate a trade‐off. Loadings that were close to zero were interpreted to indicate that the variable is largely independent of that PC. All terms were averaged by yearly responses. Transformations of variables were the same as those used in the mixed models, and bee and flower fly abundances were log (ln + 1) transformed to stabilize their variances. Animal‐pollinated flower production included species not from the seed mix. Analyses were carried out using “Proc Factor” in SAS 9.4.

We used a confirmatory SEM analysis (Grace, 2006) to test a priori predictions about the simultaneous provision of multiple ecosystem services. We tested whether the exogenous variable seed‐mix functional group diversity was a direct predictor of four endogenous variables: ANPP, butterfly, bee, and flower fly abundance. We also tested for indirect effects of seed mix functional group diversity on these four variables through its effects on volunteer biomass and animal‐pollinated flower production. Our model was based on our prediction of a potential trade‐off between invasion resistance and animal‐pollinated flower production. All terms in our SEM were as described in the factor analysis. Analyses were carried out using “Proc Calis” in SAS 9.4.

## RESULTS

**FIGURE 2:**
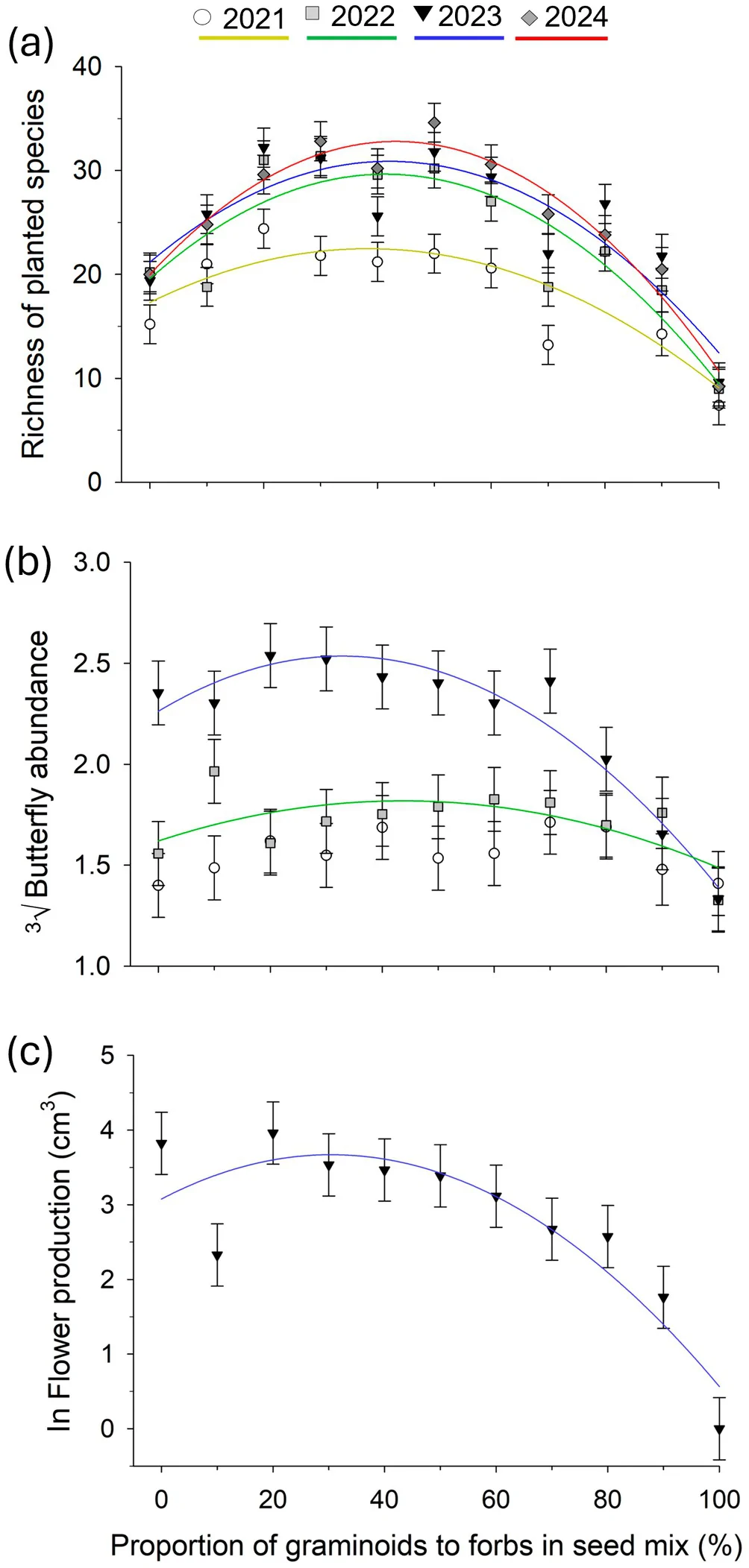
The effect of grass:forb seeding ratio (*X*) on ecosystem services (*Y*) according to year of study (significant grass:forb ratio by year interaction, see Table 2). A significant relationship is indicated by a trend line (see Table 2). Plot symbols are least squares means ± SE per year in the 900‐m^2^ plots (a and b) or in 3.14‐m^2^ sampling plots (c). Flower production from 2021 to 2022 is presented in Soley and Wilsey (2025).

**FIGURE 3:**
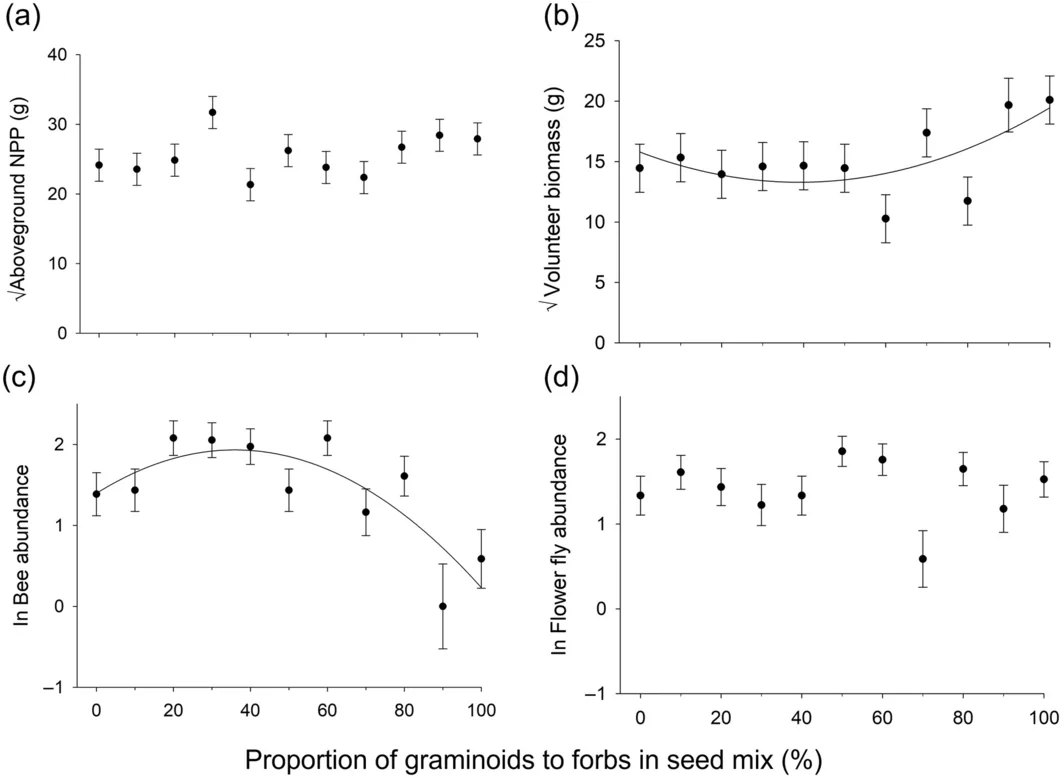
The effect of grass:forb seeding ratio (*X*) on ecosystem services (*Y*). A significant relationship is indicated by a trend line (see Table 2). Plot symbols are least squares means ± SE per square meter (a and b) or per 3.14 m^2^ (c and d). NPP, net primary productivity.

Supporting, regulating, and aesthetic ecosystem services varied significantly among seed mix grass:forb ratios (Table 2; Figures 2 and 3). Supporting services that varied were richness of planted species and animal‐pollinated flower production (Table 2), which were hump‐shaped in their response to grass:forb seeding ratio (quadratic coefficient: richness of planted species = −15.9 ± 1.4 SE, animal‐pollinated flower production = −1.32 ± 0.38 SE; Table 2). Regulating services that varied were invasion resistance and bee pollination (Table 2). The relationship between grass:forb seeding ratio and invasion resistance and bee pollination was significantly quadratic (quadratic coefficient: volunteer biomass = 4.84 ± 2.0 SE, bee abundance = −1.22 ± 0.30 SE; Table 2). Grass:forb seeding ratio had a significant quadratic effect on bee taxonomic richness ($\chi_{10}^{2}=18.6$, *p* < 0.05; quadratic coefficient: −0.77 ± 0.31 SE), and subsequent analysis of covariance revealed this effect was related to greater bee abundance at intermediate ratios (bee abundance term: $\chi_{1}^{2}=15.1$, *p* < 0.01; grass:forb ratio term: $\chi_{10}^{2}=5.6$, *p* = 0.85). Butterfly abundance, or aesthetic services, significantly varied (Table 2) and was hump‐shaped in its response to grass:forb seeding ratio (quadratic coefficient = −0.43 ± 0.10 SE; Table 2). The relationship between grass:forb seeding ratio and plant biodiversity, animal‐pollinated flower production, and butterfly abundance significantly varied across years (Table 2), which was due to an increasingly significant quadratic effect with time (Table 2; Figure 2). Grass:forb seeding ratio did not significantly affect ANPP, flower fly abundance (Table 2), or flower fly taxonomic richness ($\chi_{10}^{2}=3.6$, *p* = 0.96). We recorded a total of 271 bees and 233 flower flies (Appendix S1: Table S2) and 1039 butterflies.

**TABLE 2:** The effect of grass:forb seeding ratio on ecosystem services.

Effect	Richness of planted species	Animal‐pollinated flower production	Aboveground net primary productivity	Volunteer biomass	Bee abundance	Flower fly abundance	Butterfly abundance
Grass:forb seeding ratio	**21.8** (10, 43.1)	**5.3** (10, 43)	1.7 (10, 43)	**2.1** (10, 43)	**34.3** (10)	5.0 (10)	**3.1** (10, 38.4)
Year	**43.8** (3, 95.7)	**113.3** (2, 86)	1.1 (2, 86)	**41.8** (2, 86)	…	…	**57.4** (2, 49.8)
Grass:forb × Year	**2.2** (30, 108)	**2.4** (20, 86)	1.5 (20, 86)	0.8 (20, 86)	…	…	**1.9** (20, 62.9)
Treatment contrasts							
Linear	**48.2** (1, 43.1)	**27.4** (1, 43)	1.3 (1, 43)	3.6 (1, 43)	**14.3** (1)	0.0 (1)	**10.2** (1, 38.4)
2021	**20.4** (1, 121)	**11.4** ^a^ (1, 62.1)	…	…	…	…	0.1 (1, 119)
2022	**31.0** (1, 121)	**21.6** ^a^ (1, 62.1)	…	…	…	…	0.7 (1, 119)
2023	**23.0** (1, 121)	**38.6** (1, 62.1)	…	…	…	…	**32.3** (1, 119)
2024	**26.1** (1, 121)	…	…	…	…	…	…
Quadratic	**137.9** (1, 43.1)	**12.1** (1, 43)	0.4 (1, 43)	**5.9** (1, 43)	**16.3** (1)	0.0 (1)	**17.4** (1, 38.4)
2021	**30.0** (1, 121)	3.1^a^ (1, 62.1)	…	…	…	…	2.6 (1, 119)
2022	**83.3** (1, 121)	**10.2** ^a^ (1, 62.1)	…	…	…	…	**3.7** * (1, 119)
2023	**72.7** (1, 121)	**20.1** (1, 62.1)	…	…	…	…	**21.9** (1, 119)
2024	**113.6** (1, 121)	…	…	…	…	…	…

*Note*: Effect sizes are *F* values except for bee and flower fly abundance χ^2^ values and are presented with df in parentheses. Treatment contrasts tested linear and quadratic relationships between grass:forb seeding ratio and ecosystem services. A significant interaction between grass:forb ratio and year was followed with contrast analyses within a growing season. Statistically significant χ^2^ and *F* values are in bold.

^a^ Results presented in Soley and Wilsey (2025).

* *p* = 0.057.

Ecosystem service provision generally increased with time over the study (Table 2). Seeded plant richness, animal‐pollinated flower production, and butterfly abundance all increased over time (Figure 2). Volunteer biomass decreased over time (Table 2); there was lower volunteer biomass in the fourth growing season (least squares mean = 9.2 ± 0.92 SE) compared to the third growing season (least squares mean = 16.3 ± 0.92 SE) (Tukey's HSD, *t*_86_ = 5.9, *p* < 0.01), and lower volunteer biomass in the third growing season than the second growing season (least squares mean = 19.9 ± 0.92 SE) (Tukey's HSD, *t*_86_ = 3.1, *p* < 0.01). Animal‐pollinated flower production was significantly greater in the fourth growing season (least squares mean = 2.78 ± 0.13 SE) compared to the second (*t*_86_ = 15.0, *p* < 0.01) and third seasons (*t*_86_ = 5.9, *p* < 0.01) reported in Soley and Wilsey (2025). ANPP did not differ among years (Table 2).

Increases in seed mix diversity among grass:forb ratio treatments significantly explained greater service provision (Table 3). Increasing seed mix richness explained increases to the supporting services plant richness and animal‐pollinated flower production and the aesthetic service butterfly abundance. Increasing seed mix functional group diversity explained greater invasion resistance (lower volunteer biomass). Bee pollination services were best explained by seed mix species diversity (Simpson's 1/*D*). ANPP and flower fly abundance were not related to the changes in seed mix diversity.

**TABLE 3:** Results from generalized linear model analyses of the relationship between ecosystem services and seed mix components.

		Predictors									
		Species richness		Evenness		Simpson's 1/*D*		Functional group diversity		Forb density	
	Response variables	$\chi_{1}^{2}$	AIC	$\chi_{1}^{2}$	AIC	$\chi_{1}^{2}$	AIC	$\chi_{1}^{2}$	AIC	$\chi_{1}^{2}$	AIC
Supporting services	Richness of planted species	**54.8**	316.0*	1.0	369.8	**19.1**	351.7	**32.4**	338.5	**10.8**	360.0
	Animal‐pollinated flower production	**18.3**	427.7*	1.5	444.4	**13.7**	432.3	0.6	445.4	**16.5**	429.5
	Aboveground net primary productivity	0.2	770.9	0.0	771.1	0.1	771.0	0.2	770.9	0.7	770.4
Regulating services	Volunteer biomass	**4.0**	704.0	0.1	707.9	3.0	705.0	**5.9**	702.1*	2.6	705.4
	Bee abundance	**7.6**	277.2	0.8	284.0	**10.5**	274.4*	**8.7**	276.2	**6.4**	278.4
	Flower fly abundance	0.0	283.0	0.0	283.0	0.0	283.0	0.1	283.0	0.0	283.0
Aesthetics	Butterfly abundance	**21.6**	253.8*	0.3	275.1	**10.3**	265.1	**11.1**	264.3	**8.8**	266.6

*Note*: Statistically significant χ^2^ values are in bold. Asterisks note the lowest Akaike information criterion (AIC) among significant predictors.

The factor analysis found few or no ecosystem service trade‐offs and that most ecosystem services were correlated with species richness and diversity on PC axis 1 (Table 4). There were positive relationships among species richness and diversity and all ecosystem service variables except ANPP and flower fly abundance in the first PC, which accounted for 37% of the variance (Table 4). The second PC, which accounted for 18% of the variance, was largely a species evenness axis, with all ecosystem services having loadings close to zero (*r* values < 0.25). The third PC, which accounted for 12% of the variance, had high positive correlations by ANPP and volunteer biomass, and negative correlations with flower fly abundance on this axis (Table 4). Taken together, this analysis suggested that ANPP was independent of but not negatively correlated with other ecosystem services.

**TABLE 4:** Variable loadings onto principal components (PCs) from factor analysis of seed mix components and ecosystem services (ES).

		Variable loadings		
		PC 1 (4.49, 0.37), high richness, high ES's	PC 2 (2.21, 0.18), high evenness and forbs, low FG diversity	PC 3 (1.45, 0.12), high ANPP, volunteers, low flower flies
Seed mix components	Species richness	0.79	−0.41	0.20
	Species evenness	0.00	0.93	−0.13
	Simpson's diversity	0.76	0.59	0.05
	Functional group diversity	0.57	−0.58	0.09
	Forb density	0.71	0.62	0.01
Supporting services	Richness of planted species	0.81	−0.19	0.01
	Animal‐pollinated flower production	0.72	0.08	0.16
	Aboveground net primary productivity	−0.20	0.08	0.65
Regulating services	Volunteer biomass	−0.51	0.21	0.66
	Bee abundance	0.61	0.10	0.07
	Flower fly abundance	0.18	−0.05	−0.66
Aesthetics	Butterfly abundance	0.75	−0.09	0.23

*Note*: Associated eigenvalues followed by proportional variance explained by PCs are given in parentheses, and the values below this represent correlations between a variable and a PC.

Abbreviations: ANPP, aboveground net primary productivity; FG, functional group.

**FIGURE 4:**
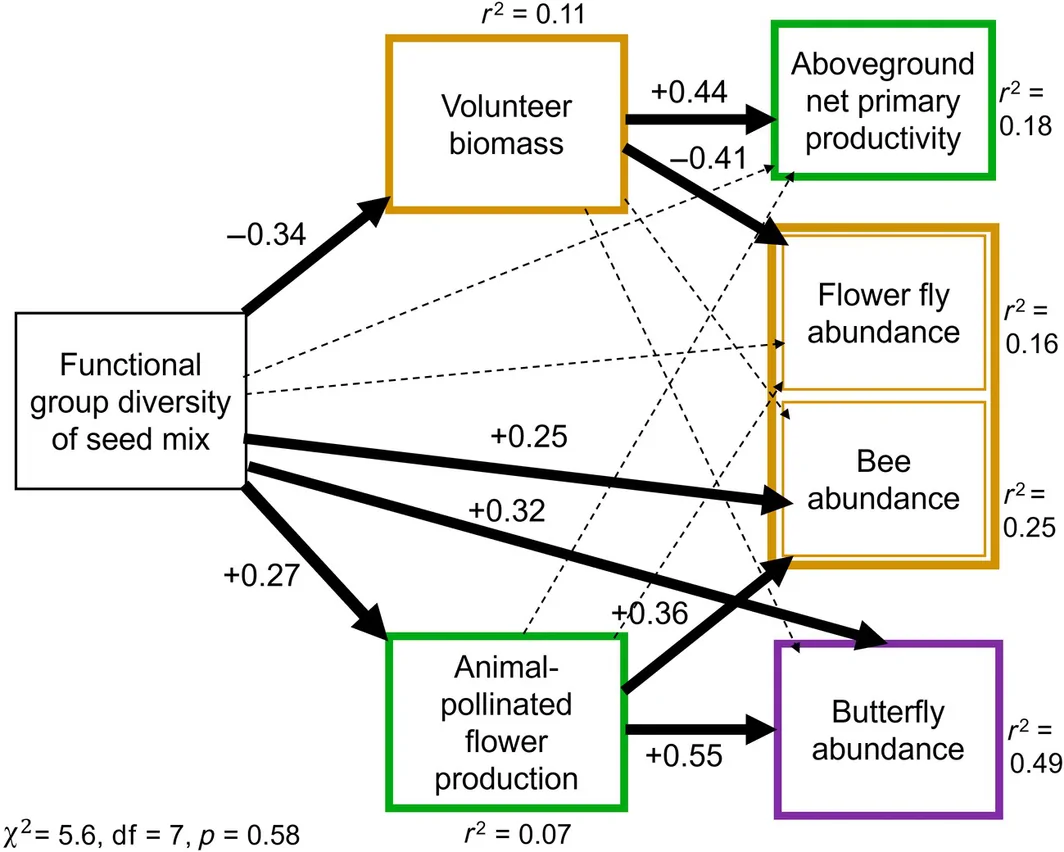
Structural equation model of the direct and indirect effects of seed mix functional group diversity on ecosystem services. Green boxes represent supporting services, gold boxes are regulating services, and the purple box is an aesthetic service. Bold paths indicate significant effects, dotted lines indicate nonsignificant effects, and numbers with paths are standardized path coefficients.

We used seed mix functional group diversity as the exogenous variable in our SEM and found it was a significant predictor of multiple ecosystem services, both directly and indirectly (Figure 4). Seed mix functional group diversity directly predicted greater animal‐pollinated flower production (0.27), lower volunteer biomass (−0.34), and higher bee (0.25) and butterfly abundance (0.32). A smaller indirect effect on bee abundance was found via functional group diversity affecting animal‐pollinated flower production, which in turn affected bees (net indirect effect 0.27 × 0.36 = 0.10). The significant indirect effect of functional group diversity via flower production on butterflies was similarly small (0.27 × 0.55 = 0.15). Volunteer biomass positively affected ANPP, so seed mix functional group diversity indirectly reduced ANPP (Figure 4).

## DISCUSSION

This study shows that intermediate grass:forb seeding ratios create vegetation of greater ecosystem services when considering the services studied here. There was a peak in supporting, regulating, and aesthetic services at intermediate grass:forb seeding ratios, which grew stronger for some services with time, while plant productivity did not significantly differ. These findings suggest there are no major trade‐offs among services when seed mix diversity is highest and that trade‐offs are more likely to occur with very high dominance of grass or forbs. Most diversity indices of seed mixtures were significantly related to increases in service provision. Seed mix diversity predicted multiple ecosystem services simultaneously by creating habitat for important flower‐visiting insects through increases to floral resources and reductions in invading plants (SEM model, Figure 4). Restorationists should emphasize grass:forb seeding ratios of greater seed functional group diversity when a major goal is to maximize ecosystem service provision after establishment.

This study builds upon previous studies of grass:forb seeding ratio by testing a wide range of ratios from 0% to 100% graminoids, which tested our prediction of a quadratic relationship between grass:forb seeding ratio and multiple ecosystem services. Our design also tested an alternative hypothesis that increasing forb proportions linearly increases pollinator abundance and richness, which was not supported. The peak in plant richness at intermediate grass:forb seeding ratios contrasts somewhat from a study in Kansas, where the lowest ratio resulted in the greatest plant biodiversity (Dickson & Busby, 2009). That experiment had lower exotic weed pressure than our study, which plays a role in reducing forb persistence in restorations (Grman et al., 2021; Kaul & Wilsey, 2021; Rinella et al., 2023). Another study in Kansas and Illinois found that plant biodiversity increased with progressive lowering of five levels of grass:forb seeding ratio (Klopf et al., 2014), but ratios below 20:80 were not part of that design. Our study shows plant biodiversity, which is driven by forb abundance in tallgrass prairie, was inhibited at the lowest grass:forb seeding ratios. Forb‐forb competition may have factored into this effect (Wilsey & Stirling, 2007), or forb establishment may be facilitated by an indirect effect of grass competition with weeds (Wilsey, 2021). This latter scenario may depend on local weed abundance, and there may be a shift to competitive native grasses as predictors of forb establishment in less weedy grasslands (Ratajczak et al., 2022). The perennial prairie species from our mixes established more readily over 4 years with intermediate grass:forb seeding ratios, which coincided with increasing animal‐pollinated flower production and butterfly visitation in these plots as well. With regard to high grass:forb seeding ratios, this study demonstrates their negative impact on plant biodiversity, as also shown for the highest ratio tested in Dickson and Busby (2009) and Klopf et al. (2014).

Our work further shows how other ecosystem services (animal‐pollinated flower production, pollination, invasion resistance, aesthetics) decline with higher proportions of graminoid seeds in restoration seed mixes. Interestingly, the highest grass:forb seeding ratios were highly invaded by volunteers in this study, which agrees with other studies (Klopf et al., 2014; Losure et al., 2007) and suggests that planting especially high grass:forb ratios may require more weed management during and shortly after restoration establishment.

Our results suggest ANPP was not significantly enhanced with grass dominance nor compromised by the presence of native forbs and their flower production. This is consistent with research showing higher ANPP can be associated with greater animal‐pollinated flower production and increases in pollinator visitation (Bennett et al., 2014; Zirbel et al., 2019). The finding of no major trade‐offs among ecosystem services at intermediate grass:forb seeding ratio is counter to studies showing a cost of some services to forage production in biodiverse grasslands (Balfour et al., 2025; Lindborg et al., 2023; Liu et al., 2025; Tallowin & Jefferson, 1999; White et al., 2004). Our estimate of forage, ANPP in a tallgrass prairie restoration, is an adequate measure of livestock forage production (Isbell & Wilsey, 2011; Owensby et al., 2008), and results suggest that its variation is independent of grass:forb seeding ratio. This finding differs from a study in Hungary that found that five levels of grass:forb seeding ratio affected ANPP, with productivity increasing as the grass proportion increased across treatments (Sonkoly et al., 2019). The only indication of a trade‐off in our study was the finding that volunteer plants contribute to plant productivity, but cause reductions in pollination services from flower flies (Figure 4). In our experiment, the forbs were almost all perennial species with relatively high biomass compared to grasses (unpublished data), and further research should be done on the grass‐forb biomass differences in this system versus others.

Our estimation of ecosystem services from different flower‐visiting insects revealed strong congruence in bee and butterfly visitation across grass:forb seeding ratios, but weaker congruence between bees and flower flies. Other ecosystem service studies showed congruence in animal visitation patterns to native plantings in North America (Bennett & Gratton, 2013; Schulte et al., 2017; Werling et al., 2014), and our study builds upon these by providing further insight into the factors that can cause such congruence. Results from our SEM suggest that bee and butterfly dependency on floral resources drove their congruence. This contrasts with incongruent patterns of bee and butterfly visitation in remnant prairie habitat (Davis et al., 2008). Bees and flower flies were weakly congruent because flower fly visitation was related to lower volunteer plant invasion and not to flower abundance, revealing divergent paths to pollination services from each guild. Further measurements beyond the fourth growing season studied here should be conducted to test if these trends continue.

Many of the diversity indices of grass:forb seeding ratio were significant predictors of ecosystem service provision and were variable in terms of their strength. Richness increases were most often associated with increasing service delivery, but it is important to note that these increases involved functional group differences—10 species grass mix, 48 species forb mix, and 58 species grass‐forb mixes—that show the transition from grass‐ and forb‐only mixes to grass‐forb mixtures had a strong positive outcome on service delivery. Management should stress increases to both graminoid and forb species richness when creating more species‐rich seed mixtures, including in cases where animal‐pollinated flower production for pollinators is a main objective. A recent large‐scale survey of grassland restorations in the United States found seed mix richness did not influence bee visitation (Simanonok et al., 2022), and our results suggest that Simpon's diversity and functional group diversity of seed mixtures may be more important for bee pollination services. Functional group diversity was the key component of the seed mix related to invasion resistance in this study. Overall, the synergistic effect of supporting and regulating services arising from diverse grass:forb seed mixes increases the aesthetics of plantings, which may feedback to peoples' well‐being to influence further investment in grassland restoration (Lundberg et al., 2019; Metrick & Weitzman, 1996).

An important caveat to mention is that several important longer term services, including soil carbon sequestration (Wilsey et al., 2020; Yang et al., 2019) and nutrient cycling, await assessment and may have different relationships with grass:forb seeding ratios. The higher C:N ratio of C_4_ grass roots suggests a positive effect of their abundance on carbon sequestration (Baer et al., 2002), but a study in tallgrass prairie restorations suggests that mixed grass‐forb communities more effectively sequester carbon in the long term (Ampleman et al., 2014). Experimental evidence shows that diverse grass communities have greater root production than communities of other plant families (Podzikowski et al., 2023). With regard to nitrogen retention, functional group diversity at intermediate grass:forb seeding ratios may optimize nutrient utilization as suggested from results with higher diversity seed mixes (Klopf et al., 2017), but this needs to be tested.

## CONCLUSIONS

This study shows that the common management decision of how much grass versus forb seed to include in seed mixtures affects the ecosystem services of resulting vegetation. Intermediate grass:forb seeding ratios create biodiverse restorations of more sustainable aesthetics compared to especially low or high ratios. Although forb seed density is often stressed for restoring pollinator habitat, seed mix functional group diversity, which is highest at a 50:50 grass:forb seeding ratio, should be emphasized more for restoring pollinator habitat in tallgrass prairie. We recommend that grass:forb seeding ratios of between 30:70 and 60:40 will be best at providing supporting, regulating, and cultural ecosystem services. Prioritizing intermediate grass:forb seeding ratios can better meet general restoration goals of enhancing biodiversity and minimizing trade‐offs between ecosystem services, which will likely satisfy more stakeholders.

## CONFLICT OF INTEREST STATEMENT

The authors declare no conflicts of interest.

## Supporting information


Appendix S1.

## Data Availability

Data (Soley et al., 2026) are available in Dryad at https://doi.org/10.5061/dryad.cfxpnvxnp. Code (NathanSoley, 2026) is available in Zenodo at https://doi.org/10.5281/zenodo.21861391.
