# Supplementary material for: Seed mixes of intermediate grass:forb ratio create vegetation of greater ecosystem service provision in restorations
Source: Ecol Appl. 2026 Sep 8;36(6):e70299. doi: 10.1002/eap.70299 (PMC13551067; doi:10.1002/eap.70299)
Supplement: Supplementary file 1 [file EAP-36-e70299-s001.pdf]

Appendix S1

Seed mixes of intermediate grass:forb ratio create vegetation of greater ecosystem service provision in restorations

Nathan Soley, Simone Schmidt, Brian Wilsey

Ecological Applications

Table S1. Species included in the grass and forb seed mixes and their abundance using the 50: 50 mix as an example. The pollination mode includes whether plants are wind or animal pollinated, and clonality is whether plants are non-clonal or clonal, producing ramets through rhizomes (Rhiz.) or stolons. Bunch grasses (cespitose grasses) are also called tussock or tufted grasses. All species in the seed mix were perennial except *Rudbeckia hirta*, a biennial. All species except *Veronicastrum virginicum* have been observed at least once at the site during the first 6 growing seasons. Seeds were cleaned to remove weed seeds and loose debris before mixes were created (i.e., had ~100% purity), and all species had high viability from a tetrazolium test.

Scientific Name	Seeds/ feet^2	Seeds/ m^2	Seeds/ plot	Pollination	Clonality	Seed viability (%)
Graminoids, Order *Poales* (Grass Mix)						
Andropogon gerardii	2	22.2	20,000	wind	Rhiz.	99
Bouteloua curtipendula	2	22.2	20,000	wind	Rhiz.	98
Carex vulpinoidea	2	22.2	20,000	wind	Bunch	99
Elymus canadensis	1.5	16.7	15,000	wind	Bunch	88
Koeleria macrantha	2	22.2	20,000	wind	Bunch	99
Panicum virgatum	2	22.2	20,000	wind	Rhiz.	98
Schizachyrium scoparium	2	22.2	20,000	wind	Bunch	98
Sorghastrum nutans	2	22.2	20,000	wind	Rhiz.	89
Sporobolus compositus (*asper*)	2	22.2	20,000	wind	Bunch/Rhiz.	95
Sporobolus heterolepis	0.5	5.6	5,000	wind	Bunch	99
TOTAL(grass)	18		180,000			
Forbs (Forb Mix)						
Amorpha canescens	0.5	5.6	5,000	animal	Rhiz.	98
Astragalus canadensis	0.5	5.6	5,000	animal	Rhiz.	98
Baptisia alba	0.1	1.1	1,000	animal	Non/Tap	93
Dalea candida	0.5	5.6	5,000	animal	Non/Tap	99
Dalea purpurea	0.5	5.6	5000	animal	Non/Tap	98
Desmodium canadense	0.5	5.6	5000	animal	Non/Tap	91
Lespedeza capitata	0.5	5.6	5000	animal	Non/Tap	98
Allium canadense	0.1	1.1	1000	animal	Bulb	90
Anemone canadensis	0.19	2.1	1,900	animal	Rhiz.	99
Artemisia ludoviciana	0.5	5.6	5,000	wind	Rhiz.	99
Asclepias incarnata	0.5	5.6	5,000	animal	Rhiz	95.
Asclepias syriaca	0.19	2.1	1,900	animal	Rhiz.	97
Asclepias tuberosa	0.1	1.1	1,000	animal	Rhiz.	96
Arnoglassum atriplicifolium	0.1	1.1	1,000	animal	Rhiz.	99
Coreopsis palmata	0.1	1.1	1,000	animal	Rhiz.	NA
Echinacea pallida	0.5	5.6	5,000	animal	Non/Tap	99
Erynigium yuccifolium	0.5	5.6	5,000	animal	Non/Tap	99
Gentiana andrewsii	0.5	5.6	5,000	animal	Non/Tap	99
Gentiana flavida (alba)	0.1	1.1	1,000	animal	Non/Tap	97
Helianthus grosseserratus	0.1	1.1	1,000	animal	Rhiz.	99
Helianthus pauciflorus	0.1	1.1	1,000	animal	Rhiz.	90
Heliopsis helianthoides	0.5	5.6	5,000	animal	Non/Tap	98
Liatris aspera	0.1	1.1	1,000	animal	Non/Corm	99
Liatris pycnostachya	0.5	5.6	5,000	animal	Non/Corm	98
Lobelia siphilitica	0.5	5.6	5,000	animal	Rhiz.	93
Monarda fistulosa	0.5	5.6	5,000	animal	Rhiz.	95
Penstemon digitalis	0.5	5.6	5,000	animal	Rhiz.	99

Phlox pilosa	0.1	1.1	1,000	animal	Rhiz.	87
Potentilla arguta	0.5	5.6	5,000	animal	Rhiz.	97
Pycnanthemum virginianum	0.5	5.6	5,000	animal	Rhiz.	99
Ratibida pinnata	0.5	5.6	5,000	animal	Rhiz.	98
Rosa arkansana	0.01	0.1	100	animal	Rhiz.	88
Rudbeckia hirta	0.5	5.6	5,000	animal	Non/Tap	98
R. subtomentosa	0.5	5.6	5,000	animal	Non/Tap	99
Silphium laciniatum	0.01	0.1	100	animal	Non/Tap	97
Solidago rigidum	0.5	5.6	5,000	animal	Non/Tap	94
Solidago nemoralis	0.5	5.6	5,000	animal	Rhiz.	95
Solidago speciosa	0.5	5.6	5,000	animal	Rhiz.	99
Symphyotrichum ericoides	0.5	5.6	5,000	animal	Rhiz.	99
S. laeve	0.5	5.6	5,000	animal	Non/Tap	94
S. novae-angliae	0.5	5.6	5,000	animal	Rhiz.	97
S. sericeum	0.1	1.1	1,000	animal	Rhiz.	97
Thalictrum dasycarpum	0.5	5.6	5,000	animal	Rhiz.	83
Tradescantia ohioensis	0.5	5.6	5,000	animal	Rhiz.	99
Verbena stricta	0.5	5.6	5,000	animal	Non/Tap	99
Veronicastrum virginicum	0.5	5.6	5,000	animal	Rhiz.	NA
Vernonia fasciculata	0.5	5.6	5,000	animal	Rhiz.	87
Zizia aurea	0.5	5.6	5,000	animal	Non/Tap	97

TOTAL(forb-legume)	18	180,000	for a 50:50 grass:forb mix
TOTAL (grass)	18	180,000	for a 50:50 grass:forb mix
	36	360,000	

Common volunteers (not in seed mix)

Cirsium arvense	animal	Rhiz.
Taraxacum officinale	apomixis	Non/Tap
Conyza canadensis	selfing	Non/Annual
Melilotus alba	animal	Non/Annual
Trifolium pratense	animal	Stolon.
Trifolium repens	animal	Stolon.
Setaria spp.	wind	Non/Annual
Thlaspi arvense	animal	Non/Annual
Aster pilosus	animal	Rhiz.
Elymus repens	wind	Rhiz.
Poa pratensis	wind	Rhiz.

Table S2. Total abundance of bee and flower fly genera observed during the study. Genera are listed below taxonomic family.

Taxa	Number observed
**Bees (Apoidea)**	
Andrenidae	
Andrena	6
Calliopsis	4
Apidae	
Apis mellifera	6
Bombus	21
Melissodes	29
Svastra	2
Colletidae	
Hylaeus	6
Halictidae	
Agapostemon	6
Augochlorella	43
Halictus	72
Lasioglossum	58
Nomia	5
Megachildae	
Megachile	5
Unknown	8
**Flower flies (Syrphidae)**	
Allograpta	2
Cheilosia	1
Eristalinus	2
Eristalis	98
Eupeodes	1
Helophilus	38
Sphaeophoria	13
Syritta	1
Toxomerus	69
Unknown	8
